# Energy metabolism dysfunction and therapeutic strategies for treating temporomandibular disorders

**DOI:** 10.3389/fmed.2025.1581446

**Published:** 2025-06-26

**Authors:** Wenjie Gao, Zhiheng Gao, Heting Xiao, Yuchen Qian, Rongkang Fan, Yonggang Li, Shaofeng Yang, Yanjun Yang, Yusen Qiao

**Affiliations:** ^1^Department of Orthopedics, The First Affiliated Hospital of Soochow University, Suzhou, China; ^2^Department of Orthopedics, Suzhou Ninth People’s Hospital Afffliated to Soochow University, Suzhou, China

**Keywords:** energy metabolism, temporomandibular joint, temporomandibular disorders, therapeutic strategies, metabolic dysregulation

## Abstract

Temporomandibular disorders (TMD) are prevalent and multifactorial conditions affecting the temporomandibular joint (TMJ). Recent studies have highlighted the central role of energy metabolism in their pathogenesis. This review focuses on how disturbances in glucose, lipid, and mitochondrial energy pathways contribute to TMJ dysfunction and degeneration. The TMJ’s complex structure relies heavily on a balanced energy supply to maintain its physiological functions. Disruptions in glucose metabolism lead to oxidative stress, inflammatory cytokine release, and chondrocyte damage. Similarly, altered lipid metabolism—particularly imbalances in ω-3 and ω-6 fatty acids—modifies inflammatory responses. Hormonal influences, including cholesterol and estrogen, further exacerbate joint degeneration via signaling pathways such as Notch and NF-κB. These metabolic disturbances trigger cellular senescence, impaired matrix synthesis, and structural breakdown of cartilage and bone. The review also evaluates current and emerging therapeutic strategies. Standard treatments such as NSAIDs and corticosteroids relieve symptoms but fail to address underlying metabolic dysfunctions. Promising alternatives include intra-articular growth factors, metabolic modulators targeting oxidative stress, and autophagy inducers that restore mitochondrial balance. These approaches aim to correct cellular energy imbalances and support tissue regeneration. TMD involve significant metabolic dysregulation in the TMJ. Understanding the role of energy metabolism offers new insights into disease mechanisms and potential therapies. Future research should prioritize metabolic regulation as a target for long-term and disease-modifying treatments in TMD management.

## 1 Introduction

The temporomandibular joint (TMJ) is a unique synovial joint essential for mastication, speech, and mandibular movement. It comprises several interrelated tissues, including the mandibular condyle, articular disc, synovium, joint capsule, cartilage, and associated musculature such as the temporalis and masseter muscles. These structures work together to maintain joint stability and function under complex mechanical loads.

The TMJ is innervated primarily by the auriculotemporal nerve, a branch of the mandibular division of the trigeminal nerve. This rich neural network enables precise control of jaw movement and transmits pain and proprioceptive signals. Blood supply is provided by branches of the superficial temporal and maxillary arteries, supporting the metabolic demands of the joint tissues.

**Figure 1:**
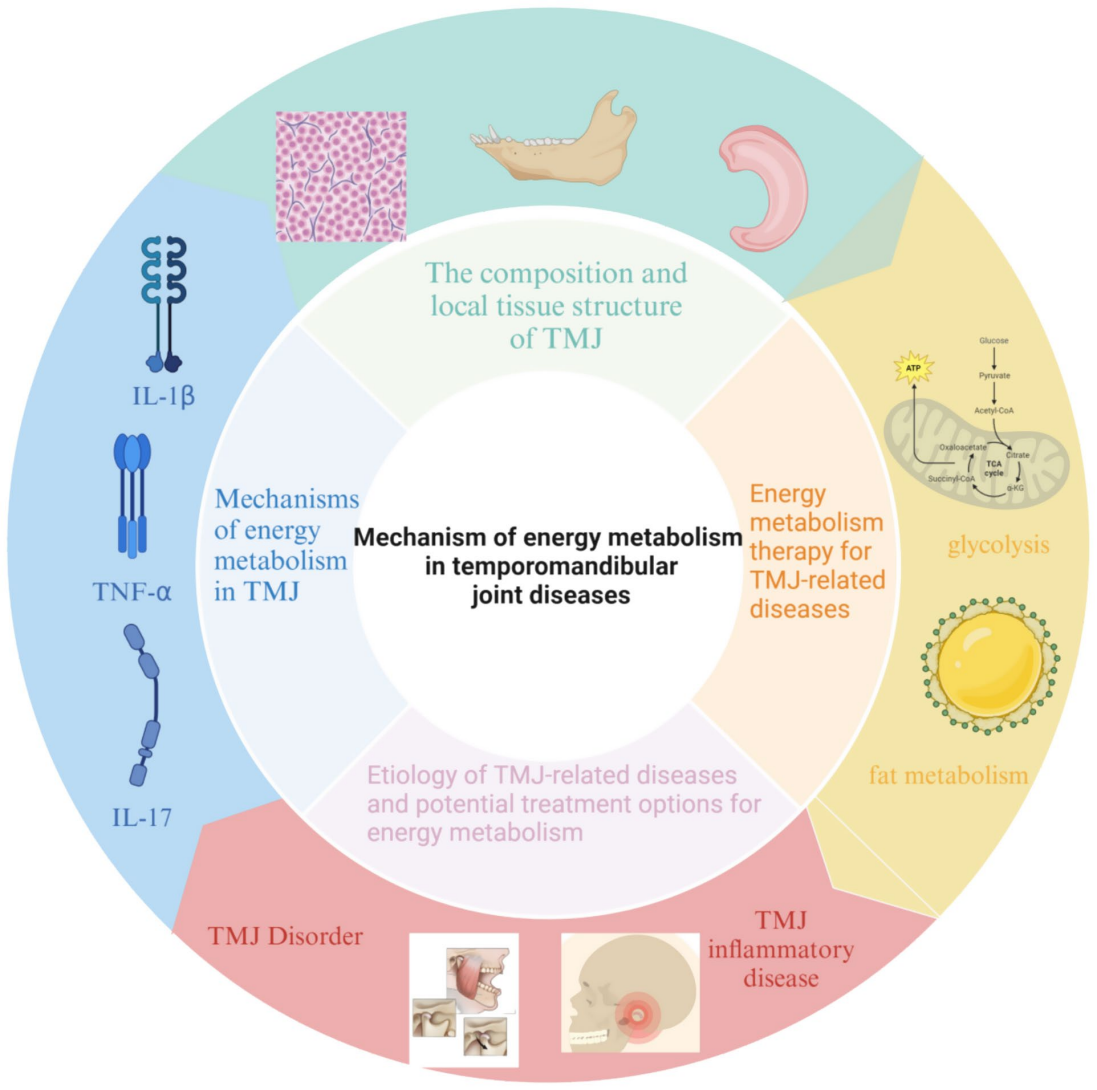
Overview of the effects of energy metabolism on the TMJ.

Multiple intrinsic and extrinsic factors affect TMJ homeostasis. These include mechanical forces from mastication, systemic inflammatory mediators, hormonal changes, and metabolic status. Disruption of any of these elements can compromise energy metabolism within the joint, leading to inflammation, matrix degradation, and degeneration of the joint structures. These interconnected mechanisms are summarized in Figure 1, which provides an overview of the effects of energy metabolism on the TMJ.

## 2 Composition of the TMJ and local organization of the joints

The TMJ is a complex joint composed of four types of tissues: bone, ligaments, cartilage, connective tissue, and the joint capsule. The maintenance of its normal structure depends on the coordinated function of these tissues. The TMJ is subdivided into several regions, with the temporalis muscle, masseter muscle group, joint capsule, and ligaments being the most prominent structures. Additionally, various other components are present within the TMJ, including the synovium, joint capsule, and cartilage. The synovium is a connective tissue lining the joint cavity that secretes synovial fluid, which prevents infection and absorbs inflammation (1). The joint capsule is a fibrous connective tissue structure covered by a synovial membrane that secretes joint fluid and helps cushion pressure (2). Articular cartilage consists of a matrix of collagen and chondroitin sulfate, which maintains joint pressure balance, protects joint surfaces, and promotes synovial fluid production. These tissues form a complex structural network that supports the normal structure and function of the TMJ. Abnormal energy metabolism in the TMJ is linked to many diseases, with complex mechanisms underlying their development.

### 2.1 Composition of the TMJ

The TMJ consists of various tissues, including the temporalis muscle, masseter muscle group, joint capsule, and ligaments. The temporalis muscle is a primary component of the TMJ, with fibers divided into two major types: type I fibers (yellow fibers) and type II fibers (red fibers). Type II fibers are further categorized into long helical myosin, which has high elasticity and strength, and short-chain actin, which has strong mechanical sensitivity. Both fiber types work in tandem to maintain the normal position of the articular disc and absorb pain signals in the event of TMJ injury. The masseter muscle group, composed of masticatory muscle fibers, is the largest tissue contributing to the TMJ, playing key roles in chewing, speech, and swallowing (3). The joint capsule, made of collagen fibers, functions to regulate joint pressure, maintain stability, and protect joint surfaces. The joint capsule is unique compared to other tissues in the TMJ due to its composition, which closely resembles that of the TMJ itself (4).

### 2.2 Local organization of the joint

The TMJ is anatomically composed of four tissues and features a complex structure, with the joint capsule and synovium being key components. The movement of the TMJ is accomplished by both the muscles and the joint capsule, with intricate interactions between the two. The masseter muscle group is responsible for masticatory movements, directly connecting with the temporalis muscle, while the connection between the temporalis muscle and the joint capsule is indirect. The joint capsule and synovium play essential roles in secreting and absorbing synovial fluid and cushioning pressure. Together with tissues such as bone, cartilage, and ligaments, they form the complex structure of the TMJ (3).

Histologically, the TMJ is composed primarily of the articular disc, articular fossa, articular capsule, and articular disk. Dense fiber bundles are present in the connective tissue surrounding the articular disc, effectively restricting its movement. Additionally, the TMJ capsule contains a layer of collagen fiber bundles that are flexible and absorb pressure. The articular disc is located within the joint capsule and is attached to the lateral margin of the joint capsule. It is composed primarily of collagen and elastin fibers. Within the joint capsule, the articular disc forms a capsule-like structure made of connective tissue, located on the inner surface of the joint cavity. This regularly arranged meniscus effectively restricts the motion of the TMJ. Surrounding the articular disc is a fibrous ring composed of collagen fibers known as the fibrocartilaginous ring. Collagen fibers are critical for maintaining the structure and function of the TMJ. Collectively, these tissues form a complex, fine-tuned, and structurally intact system.

## 3 Temporomandibular disorders and their etiology

Temporomandibular disorders (TMD) represent a prevalent group of conditions affecting approximately 34% of the global population, with notable variation across regions—47% in South America, 33% in Asia, 29% in Europe, and 26% in North America. The prevalence is higher among adults aged 18–60 years (41%) and disproportionately affects females, potentially due to hormonal and psychosocial factors (5).

The etiology and mechanisms of TMD are multifactorial, involving: traumatic injury, TMJ muscle dysfunction, inflammatory diseases of the TMJ, degenerative lesions of the TMJ, TMJ tumors, and inadequate recovery following trauma (6). Intra-articular inflammation and cartilage degeneration, driven by pro-inflammatory cytokines and matrix degradation, are major contributors to TMD (7). Osteoarthritis, involving synovitis, cartilage loss, and bone remodeling, is a key pathological mechanism underlying TMJ inflammation and damage (8).

Numerous studies have focused on the mechanisms of energy metabolism, especially in relation to osteoarthritis of the TMJ. Significant findings have emerged from research on the skeletal system. Based on a review and analysis of the literature, the following conclusions can be drawn:

(1) Osteoblasts can stimulate osteoclastogenesis and enhance osteoclast activity by secreting bone morphogenetic proteins (BMPs), cytokines, and other factors, leading to localized bone lesions (9, 10). In the case of bone lesions in the TMJ, osteoblasts promote osteoclastogenesis by secreting inflammatory factors and chemokines, and they promote osteoclast maturation, differentiation, and activation by secreting OPG, among other factors. Osteoblasts can also degrade components of the cartilage matrix through the secretion of matrix metalloproteinases (MMPs), resulting in the loss of normal structure and function. Osteoclasts contribute to this process by secreting inflammatory factors, such as tumor necrosis factor-α (TNF-α), and growth factors that stimulate osteoclast formation and functional maturation (11).
(2) Osteoblasts also promote osteoclastogenesis and functional maturation through the secretion of TGF-β. In inflammatory diseases of the TMJ, TGF-β stimulates the proliferation and differentiation of osteoblasts while activating osteoclasts, leading to localized bone lesions (12). In degenerative diseases of the TMJ, TGF-β activates osteoblasts to secrete TGF-β1, promoting osteoclast differentiation and functional maturation (11).
(3) Fibroblasts play a role in promoting fibrosis in the TMJ by secreting collagen, proteoglycans, and other matrix components. In degenerative diseases of the TMJ, fibroblasts promote tissue repair and reconstruction by secreting these matrix components. In inflammatory diseases of the TMJ, fibroblasts synthesize large amounts of collagen and matrix components, maintaining the integrity of the joint capsule by producing hyaluronic acid, proteoglycans, collagen, and other substances (13).
(4) Fibroblast stem cells contribute to trauma repair and regeneration in the TMJ by synthesizing multiple growth factors (13).

### 3.1 Trauma

Trauma is a major cause of TMJ disorders, including fractures, joint dislocations, and ligament injuries. Trauma to the TMJ can result from mechanical stress, vibration, chemical irritation, local inflammation, and immune responses, with joint dislocations and ligament injuries being the most common outcomes. Mechanical stress can raise the incidence of TMJ dislocation from 30 to 70%, and the severity is strongly correlated with the injury site, particularly the posterior-inferior portion of the condyle, such as its posterior margin (14). The mechanism of traumatic joint dislocation is related to occlusal forces; occlusal disorders impair the movement of the condyle in the occlusal plane, altering the condylar position. Overbite of the TMJ can result in abrasion of the posterior region of the articular disc, leading to TMJ dysfunction (15).

Mechanical vibration can further damage the TMJ by applying direct mechanical stress to the anterior and posterior cartilage of the articular disc. When mechanical vibration and stress are combined, they can cause structural changes in the TMJ, such as subchondral bone resorption and osteoclastic resorption (16). Chemical stimuli also play a role in TMJ injury, contributing to post-traumatic inflammation, pain, articular disc displacement, dislocation, and muscle spasms. Local inflammation and pain can lead to TMJ dysfunction and degenerative conditions, including TMJ osteoarthritis. Traumatic inflammation can disrupt cytokine expression and initiate tissue damage, with cytokines such as IL-1β, TNF-α, and IL-17 playing a role in stimulating TMJ injury (17).

### 3.2 Temporomandibular joint muscle dysfunction

The etiology and pathogenesis of TMJ muscle dysfunction are complex and may involve several mechanisms:

(1) Muscle disease: The TMJ muscles are the primary active organs of the joint, and their dysfunction is more intricate than that of other tissues and organs. Local mechanical stress on the TMJ can lead to muscle dysfunction, which, in turn, may cause changes in the surrounding tissue structures, such as joint disc displacement and ligament damage. Dysfunction of the TMJ muscles can result in further complications, including articular disc displacement and ligament injuries (18).
(2) Periarticular diseases: Conditions affecting the tissues surrounding the TMJ, such as periarticular synovitis and osteoarthritis, can contribute to periarticular muscle dysfunction. Collagen synthase 1 (COL-1) is involved in the pathogenesis of TMJ periarticular synovitis. Studies have indicated that COL-1 plays a key role in this condition by regulating collagen synthase (COX). Osteoblasts secrete matrix metalloproteinases (MMPs), which are implicated in the development of TMJ osteoarthritis. Additionally, osteoblasts produce MMP-9 during the progression of degenerative TMJ lesions, promoting the worsening of these conditions (19).
(3) Maxillofacial trauma: Trauma to the maxillofacial area often leads to TMJ peripheral muscle dysfunction because it can damage the peripheral muscles around the joint. The primary mechanisms believed to cause maxillofacial trauma include direct injury, indirect injury, and tissue displacement. Many studies suggest that indirect injury, compared to direct injury, has a more complex and variable mechanism. In conclusion, numerous studies indicate that TMJ muscle dysfunction is closely related to these processes (18).

In addition to these factors, increasing evidence suggests excessive activity of the masticatory muscles can lead to myogenous pain, a common symptom in TMD patients. Bruxism significantly elevates the risk of developing TMD, increasing the odds by 2.25 times overall, with awake bruxism increasing the risk by 2.51 times and sleep bruxism by 2.06 times (20). Moreover, a global meta-regression study reported a 63.5% prevalence of TMD among individuals with bruxism, reaching as high as 98.3% in North America (21). These findings highlight the interactive pathophysiology between masticatory muscle dysfunction and abnormal muscle activity, suggesting that long-term bruxism may play a pivotal role in the development and progression of TMD.

### 3.3 TMJ inflammatory diseases

Temporomandibular joint inflammatory disease, also known as temporomandibular joint osteoarthritis (TMJOA), affects the TMJ and its surrounding muscles (22). The causes of TMJ inflammatory diseases are diverse and include conditions such as rheumatoid arthritis, osteoarthritis, and psoriatic arthritis. The severity of these disorders ranges from mild to severe, and trauma to the TMJ can lead to joint degeneration. If left untreated, this degeneration may result in joint fusion. In cases of inflammatory joint disease, inflammation can persist even after treatment. Diagnosis of TMJ inflammatory diseases typically involves a combination of medical history, physical examination, and imaging studies. Common diagnostic techniques include panoramic radiographs and magnetic resonance imaging (MRI) (23), with MRI being particularly valuable for assessing soft tissues. Although the biochemical evaluation of synovial fluid can improve understanding of TMJ pathophysiology, it is not yet widely used in clinical practice. Symptoms of inflammatory disease in the TMJ include pain on the affected side, limited mouth opening, and difficulty eating. The pain may originate from the joint itself, the associated muscles, or both (23, 24). Additionally, TMJ inflammatory diseases may be linked to other conditions, such as Lyme disease, which can cause a variety of inflammatory and neurological symptoms, including TMJ syndrome.

### 3.4 Degenerative lesions of the TMJ

Temporomandibular joint (TMJ) degeneration is the most common form of TMJ disease and is characterized by progressive histological changes, including the destruction, repair, reconstruction, and regeneration of cartilage and bone tissue, ultimately leading to impaired joint function. The primary drivers of TMJ degeneration are intra-articular inflammation and cartilage breakdown, which often result from biochemical imbalances and structural deterioration in the subchondral bone.

In degenerative TMJ lesions, the degradation of cartilage tissue frequently triggers changes in the local bone architecture, which subsequently cause pathological alterations in the joint capsule and articular disc, ultimately contributing to joint dysfunction. For instance, in TMJ osteoarthritis (TMJOA), chemical changes such as reduced proteoglycan synthesis, collagen fiber fragmentation, and increased expression of matrix-degrading enzymes, along with biomechanical stress, lead to pathological interactions between the articular disc and surrounding joint structures (25). These changes result in altered bone remodeling, aggravating the progression of joint degeneration.

Within the TMJ discs of TMJOA patients, many chondrocytes, mesenchymal cells with chondrocyte-like characteristics, and collagen fibers are present, reflecting the tissue’s attempt at repair but also contributing to pathological remodeling. As cartilage deteriorates, disruptions in extracellular matrix (ECM) homeostasis—such as depletion of proteoglycans, abnormal chondrocyte proliferation, and increased catabolic signaling—further destabilize the disc-bone interface, leading to additional degenerative changes (26). These bone and cartilage structural modifications, including changes in chondrocytes, chondrocyte-like cells, and collagen fibers, can initiate or exacerbate TMJOA. Moreover, the accumulation and dysregulated activity of these cells and matrix components within the disc may amplify joint deterioration and accelerate disease progression.

## 4 Energy metabolism in the TMJ: mechanisms

The TMJ is a complex structure with specialized metabolic activity that supports its functional demands. Glucose and lipid metabolism (27) are the two primary energy pathways in TMJ tissues, with glucose serving as the main energy source. Within muscle cells associated with the TMJ, glucose undergoes glycolysis and is further metabolized via the tricarbo xylic acid (TCA) cycle to produce adenosine triphosphate (ATP), the principal energy carrier (28). Phosphocreatine functions as an energy reservoir by donating phosphate groups to regenerate ATP during high energy demand, particularly in muscle (29). Key enzymes such as the pyruvate dehydrogenase complex (PDC) facilitate the conversion of pyruvate into acetyl-CoA, linking glycolysis to the TCA cycle. Lactate dehydrogenase (LDH) catalyzes the interconversion of pyruvate and lactate, contributing to energy metabolism under anaerobic conditions.

### 4.1 Involvement of sugar metabolism in TMD

**Figure 2:**
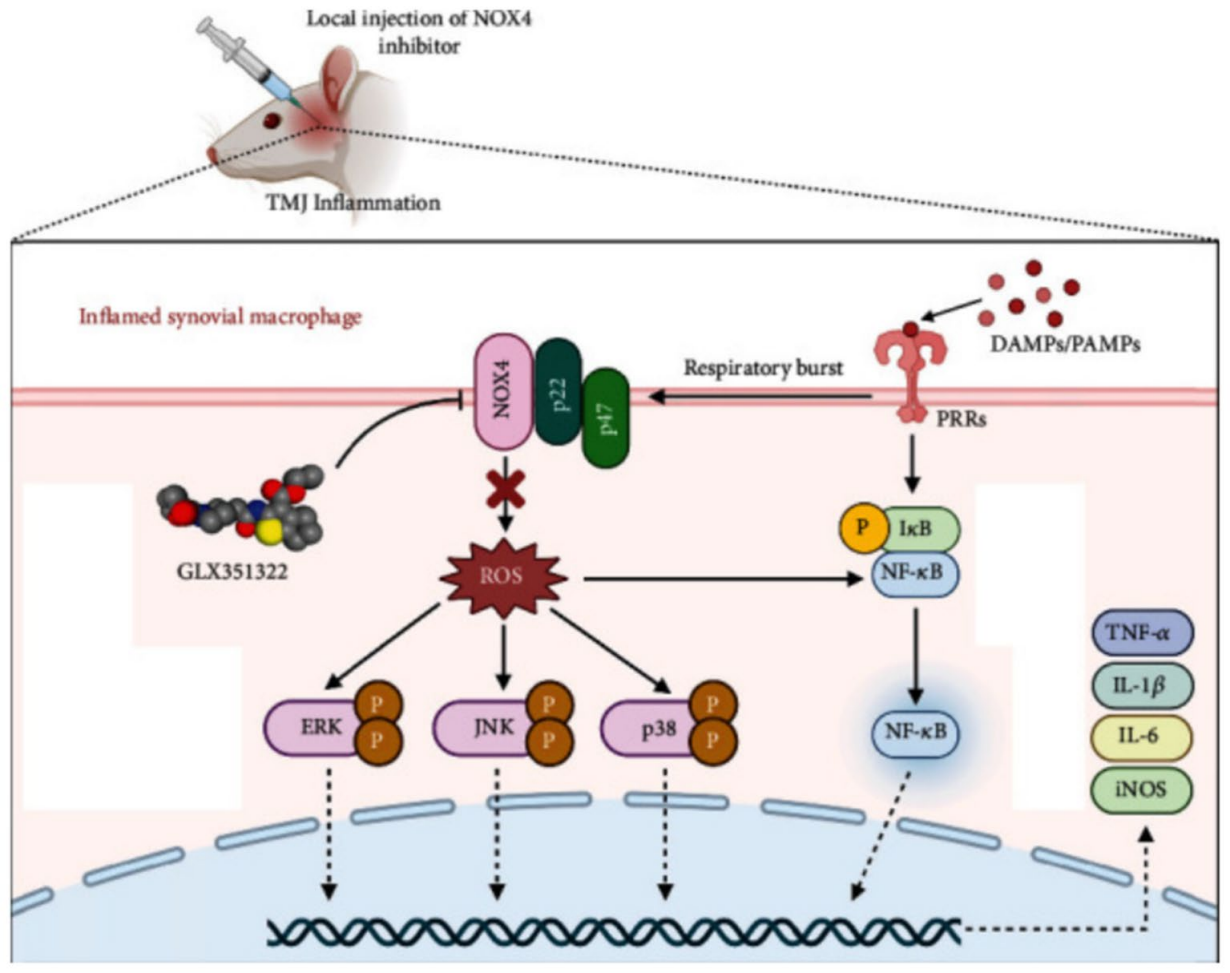
Mechanism of NOX4-mediated TMJ inflammation and therapeutic role of GLX351322.

**Figure 3:**
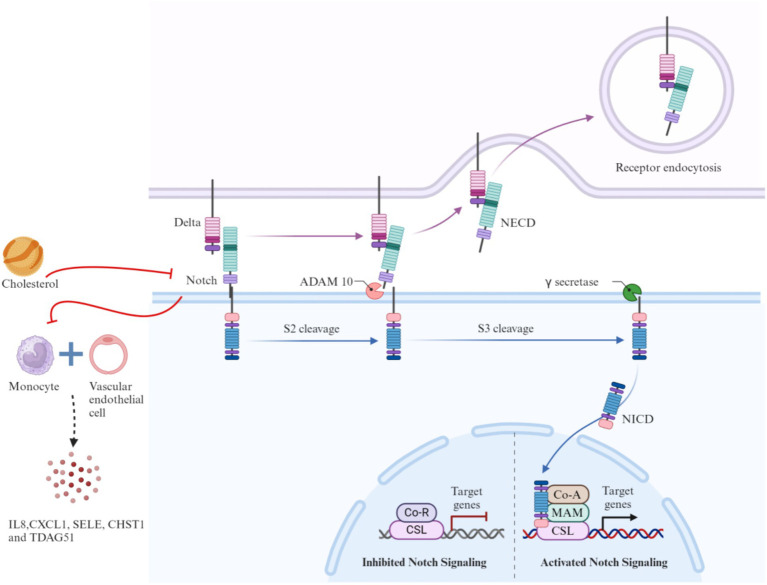
Mechanism of notch signaling activation and its modulation by metabolic and inflammatory factors.

As a mobile joint, the TMJ requires energy from its cartilage and surrounding soft tissues to maintain normal physiological functions, such as mouth opening, closing, and masticatory movements. This energy is primarily derived from the oxidative breakdown of glucose. Aerobic glycolysis plays a significant role in energy metabolism in chondrocytes, and in TMJ joints, both glucose and oxygen are critical for the survival of TMJ mesenchymal cells. Adequate glucose levels and slightly reduced oxygen concentrations may promote the growth of TMJ cells, which may be linked to oxidative phosphorylation within these cells (30). Consequently, the shift from aerobic to anaerobic environments may be crucial in studies of TMJ cells *in vitro*. This factor has been applied in subsequent research on the role of reactive oxygen species (ROS) in various forms of OA, including TMJOA (31, 32). The pentose phosphate pathway (PPP) is the primary metabolic route involved in glucose metabolism in TMJ cells. In this pathway, glucose-6-phosphate dehydrogenase (G6PD) is the key rate-limiting enzyme, coordinating the synthesis of nicotinamide adenine dinucleotide phosphate (NADPH). NADPH is essential for maintaining the cell’s reducing capacity and plays a critical role in the antioxidant defense system of TMJ cells, which includes elements such as glutathione, catalase, and superoxide dismutase. These components depend on NADPH to function properly (33). The interventional role of NADPH in regulating oxidative stress is directly linked to the inflammatory pathophysiology of the TMJ. Figure 2 illustrates this mechanism by detailing how NADPH oxidase 4 (NOX4) contributes to TMJ inflammation through ROS production and how its inhibition by GLX351322 alleviates disease progression. Recent studies have shown that GLX351322, a selective NOX4 inhibitor, effectively reduces TMJ osteoarthritis by interrupting the ROS/MAPK/NF-κB signaling cascade (34), offering a promising therapeutic avenue. Furthermore, glucose metabolism has been implicated in the treatment of TMD. Clinical trials have shown that hypertonic glucose augmentation therapy (DPT) is effective in treating TMD (35). Diabetic patients, who are generally characterized by hyperglycemia, are more likely to experience TMD, suggesting that DPT therapy may produce better outcomes in these patients (36). Although DPT shows therapeutic potential, its efficacy has yet to be conclusively validated through large-scale clinical trials, warranting further investigation. Figure 3 provides mechanistic insight into the Notch signaling pathway, which has emerged as a critical regulatory axis in TMJ inflammation and may intersect with glucose and cholesterol-related metabolic changes. This signaling cascade not only governs gene transcription related to immune and inflammatory responses but is also modulated by metabolic inputs such as cholesterol levels and cellular stress signals.

### 4.2 Fat metabolism involvement in TMD

#### 4.2.1 Anti-inflammatory potential of long-chain unsaturated fatty acids in TMD

Given the complexity of fatty acid classification, this section focuses on the effects of long-chain unsaturated fatty acids (LCUFAs) on the TMJ, while excluding saturated fatty acids (SFAs), short-chain fatty acids (SCFAs), and medium-chain fatty acids (MCFAs), as current studies have not identified significant effects from these types. Preclinical research shows that LCUFA metabolites exert opposite effects on inflammation and nociception: ω-6 (n-6) LCUFAs exacerbate, whereas ω-3 (n-3) LCUFAs alleviate, both processes (37). Animal studies further confirm the anti-inflammatory role of ω-3 LCUFAs in ovariectomy-induced temporomandibular arthritis (38), potentially through energy-consuming catabolism and modulation of IL-1β, IL-10, and TNF-α expression (39, 40). These fatty acids also promote increased fiber content and thickening of the hypertrophic condylar cartilage layer. For TMD patients, ω-3 LCUFAs help reduce joint inflammation, pain, and swelling, while supporting bone and cartilage repair—resulting in improved joint function. Due to these benefits, several studies recommend ω-3 LCUFAs as an alternative to ibuprofen.

#### 4.2.2 Cholesterol, notch signaling, and sex hormones in TMJ pathophysiology

As discussed earlier, diabetic patients with elevated cholesterol levels are at higher risk of TMD, prompting investigation into whether cholesterol impacts TMJ via energy metabolism. While current studies have yet to confirm a direct link between cholesterol and TMJ, evidence indicates that the Notch signaling pathway is essential for TMJ development and disease (41, 42). Notch influences cartilage and osteoarthritis by regulating metabolic activities in fibroblasts and chondrocytes, particularly in fibrochondral stem cell (FCSC) fate and TMJ homeostasis. Cholesterol has been shown to activate Notch signaling and promote stem cell development (43), and dietary cholesterol alters intestinal cell differentiation through Hr96-mediated Notch signaling (44), suggesting possible relevance to TMJ regulation, as shown in Figure 2. Additionally, estrogen significantly influences TMJ pathology. In animal models, estradiol (E2) enhances neural responsiveness in TMJ regions (45) and promotes fibrocartilage matrix degradation by upregulating matrix metalloproteinases MMP9 and MMP13 (46). Adolescents with fluctuating sex hormone levels—especially females—exhibit increased joint laxity and TMJ hyperactivity (47). In males, low levels of free testosterone and DHEA-S correlate with increased TMJ pain. Estrogen also contributes to sex-specific TMJ remodeling under mechanical stress and has been associated with TMD via estrogen receptor activity (48).

### 4.3 Cytokine and chemokine networks in TMJ inflammation and regeneration

Cytokines play crucial roles in cell growth, tissue remodeling, and maintaining homeostasis in the body. However, they can also mediate harmful effects such as inflammation and autoimmune responses. Numerous studies have analyzed cytokines in the synovial fluid of TMD patients, linking them to pathological mechanisms (40). In the TMJ, two key cytokines, IL-1β and TNF-α, regulate the expression of IL-6 and related cytokines (49, 50). IL-1β and TNF-α stimulate gingival fibroblasts to produce IL-6-type cytokines, such as IL-6, IL-11, and leukemia inhibitory factor (LIF). These cytokines are pleiotropic, stimulate bone resorption, and are expressed by multiple cell types (51). Specifically, IL-1β and TNF-α increase the mRNA and protein expression of IL-6 and LIF in a concentration-dependent manner, primarily through the MAP kinase signaling pathway, though not through the NF-κB pathway. This suggests that IL-1β and TNF-α influence IL-6-type cytokine expression via distinct signaling pathways, which may impact the pathological process in the TMJ. Moreover, the production of IL-6-type cytokines may affect the pathomechanism of periodontal disease through the activation of MAP kinase-mediated pathways. In summary, IL-1β and TNF-α are critical regulators in the TMJ, controlling the expression of IL-6 and related cytokines, likely through MAP kinase activation without involving NF-κB. In addition to these pathways, other chemokines such as SDF-1 and RANTES play key roles in the treatment of bone marrow mesenchymal stem cells (BMSCs) in the TMJ region. These chemokines promote the migration of BMSCs to damaged cartilage via their receptors CXCR4 and CCR1, contributing to tissue repair and regeneration. Additionally, chemokines such as MCP-1, MIP-1α, MIP-3a, RANTES, IL-8, SDF-1, and fractalkine play important roles in the progression of TMJ disease by promoting the chemotactic activity of inflammatory cells, leading to the destruction of structures such as the synovium, cartilage, and bone (52). A summary of these findings is provided in Table 1.

**Table 1:** Mechanisms of influence of several cytokines.

Class	Targeted cell	Signaling pathway	Effect/conclusion	References
MCP-1	Fibroblast-like synoviocytes	P2Y 13 purinergic receptor/ERK signaling axis	Enhanced MEK/ERK-dependent expression of MCP-1/CCL2 in TMJ FLS	Ogura et al. (77) and Yokota et al. (78)
MIP-1α	Basophil-lineage cells	CCL3	Chemotaxis	Swathi et al. (79)
MIP-3a				
RANTES	Macrophage	RANKL	DDw/oR-induced early-stage TMJ DJD	Feng et al. (80)
IL-10	Synovial fluid and synovial tissue	/	TNFalpha, IL-1, IL-6 and IL-8 were inhibited	Kacena et al. (81)
SDF-1	BMSCs	GFP-BMSC	Bone-formation	Lu et al. (82)
Fractalkine	chondrocyte	p38	Bone resorption	Guo et al. (83)

### 4.4 Hypothesized link between gene expression, molecular memory, and TMJ energy metabolism

Although previous studies have not directly explored the relationship between TMJ energy metabolism and gene expression, this connection can be inferred through the concept of molecular memory and its effect on energy expenditure during gene expression (53, 54). Molecular memory refers to a cell’s or tissue’s ability to retain a particular state after being exposed to a stimulus, and it can significantly influence the energy required for gene expression (55). This memory capacity is achieved by altering gene expression patterns, which in turn impacts the function and behavior of cells. When molecular memory is involved in gene expression, cells must expend additional energy to maintain this memory state. This is because, to sustain memory, the cell continuously replicates and adjusts associated gene expression, leading to increased energy consumption.

Applying this concept to the relationship between TMJ energy metabolism and gene expression, it can be hypothesized that physiological activities such as chewing and speaking may induce changes in the expression of specific genes to adapt to different mechanical loads and biomechanical demands. These gene expression changes may be accompanied by the formation of molecular memories, allowing cells to respond more efficiently to similar stimuli in the future. Thus, TMJ energy metabolism is not only influenced by physical and chemical factors but may also be regulated through gene expression via molecular memory. However, since direct evidence linking TMJ energy metabolism and gene expression is currently lacking, these hypotheses require further investigation to be validated.

### 4.5 Mechanotransduction and its effects on TMJ cellular and matrix homeostasis

**Figure 4:**
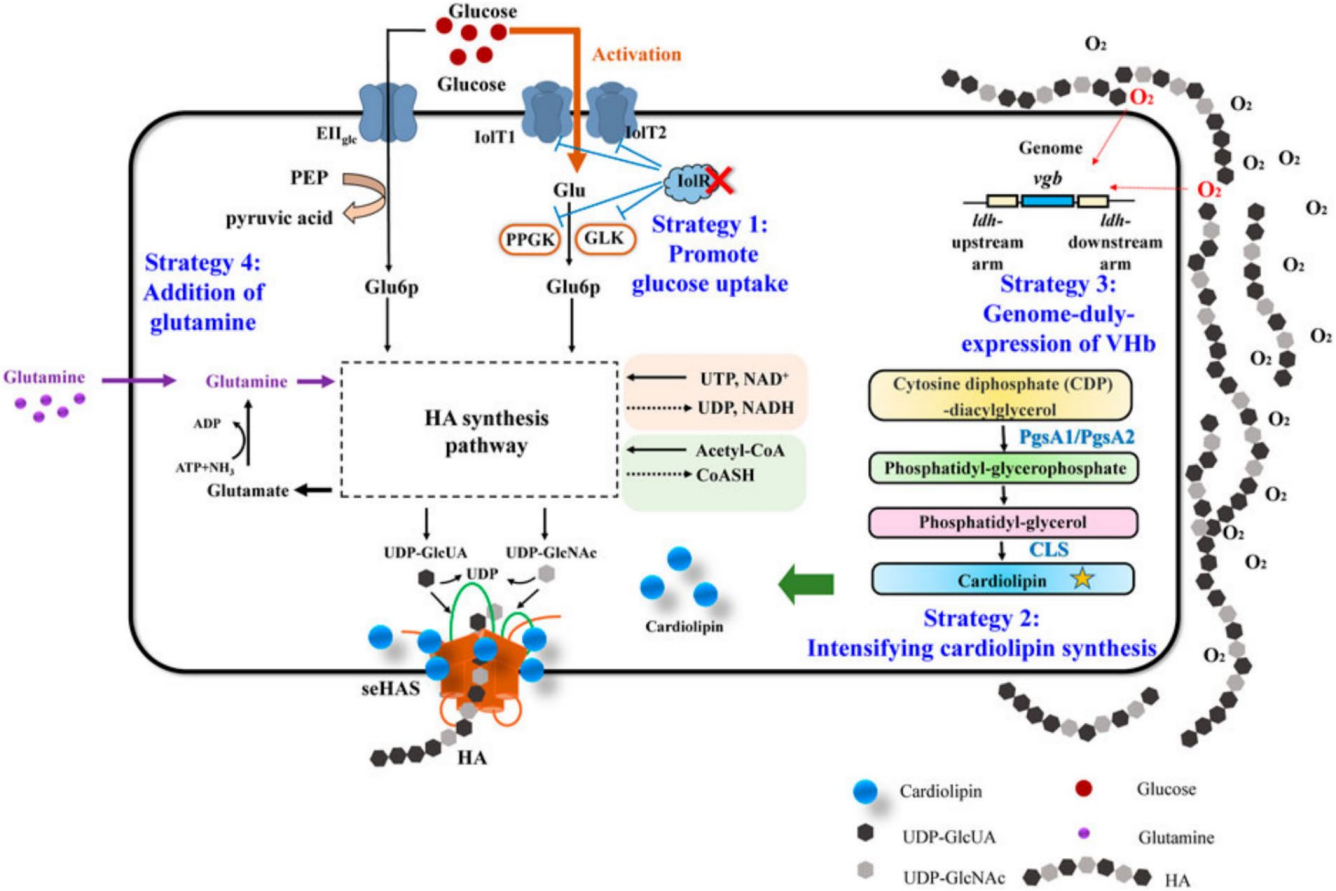
Hyaluronic acid metabolism in normal TMJ.

**Figure 5:**
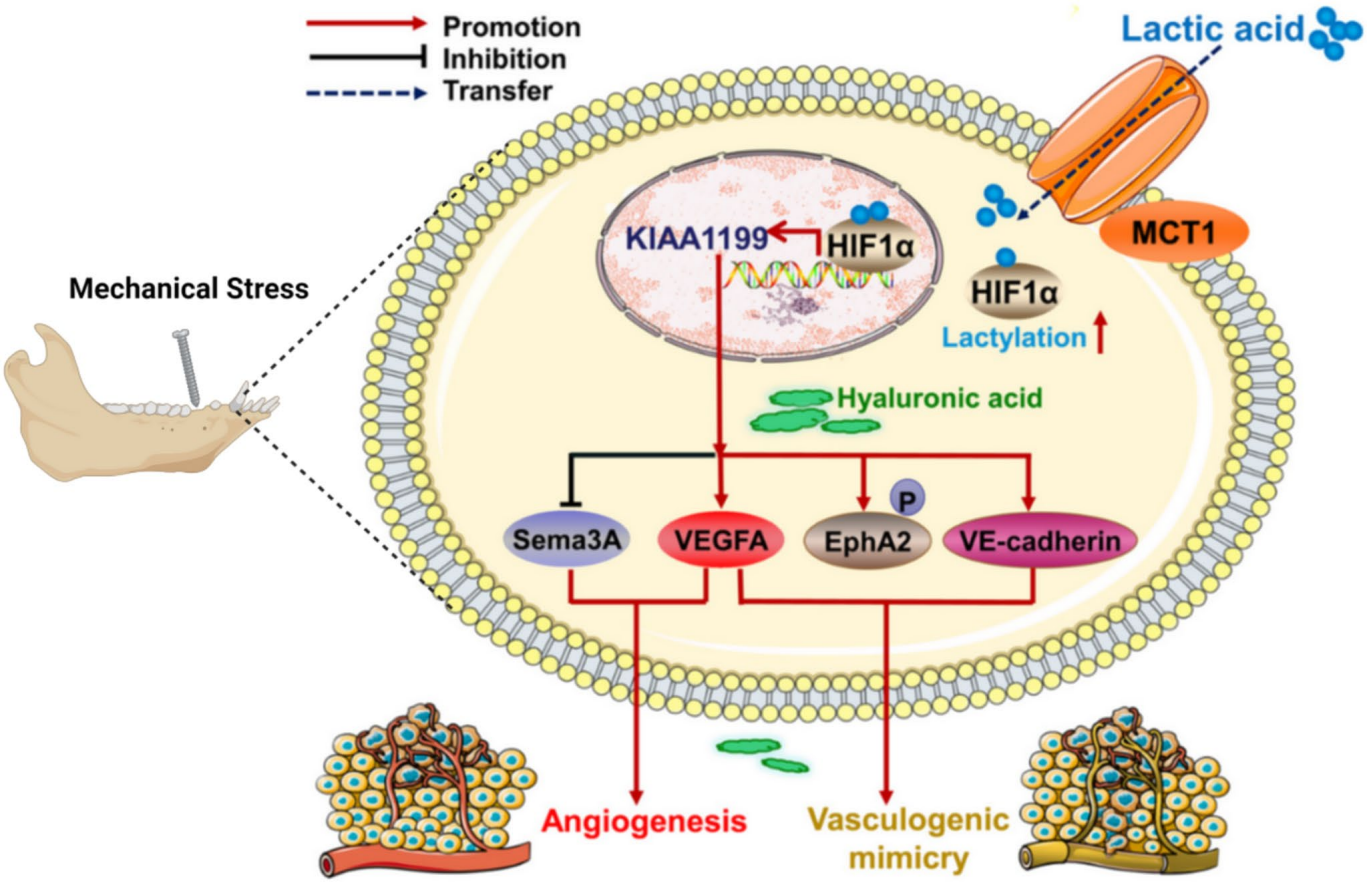
Changes of signal pathway in TMJ under mechanical stress.

The impact of mechanical loading on intracellular signaling pathways in the TMJ is primarily observed in the following areas:

1) Mechanical work and cellular responses: The amount of mechanical work in the TMJ is correlated with the type of jaw activity and is evenly distributed across the articular surface. Different jaw activities can result in varying mechanical loads, which influence intracellular signaling (56). Figures 4, 5 illustrate these complex biochemical and biomechanical interactions. Figure 4 focuses on optimizing hyaluronic acid (HA) synthesis, a key component of the TMJ extracellular matrix, while Figure 5 outlines the signal transduction pathways altered by mechanical stress in the TMJ.
2) Effect of mechanical loading on cytokine expression: Mechanical loading modulates proinflammatory signaling by attenuating TNF-α-induced MMP-13 expression, potentially via downregulation of NF-κB and AP-1 transcriptional activity, thereby reducing matrix degradation (57).
3) Effect of mechanical loading on proteoglycan expression: Mechanical stimulation alters the transcription and accumulation of key proteoglycans—particularly aggrecan and decorin—in TMJ cartilage discs. These changes affect pericellular matrix organization and influence mechanosensitive signaling pathways through receptors such as integrins and syndecans (58).
4) Mechanical loading modifies the composition and viscosity of synovial fluid, altering concentrations of lubricin and hyaluronic acid. These changes affect frictional forces on the cartilage surface, which in turn influence cellular mechanoreception and downstream signaling cascades (59, 60).
5) Solute transport: Compressive forces impair the diffusion of nutrients and signaling molecules through the cartilage matrix, potentially disrupting intracellular homeostasis and affecting signal transduction pathways reliant on paracrine factor availability (61).

## 5 Therapeutic strategies targeting energy metabolism in TMJ disorders

The treatment of TMD generally falls into two primary categories: pharmacological management, particularly for inflammatory osteoarthropathies and autoimmune conditions such as rheumatoid arthritis, and surgical interventions, especially in cases involving abnormalities in TMJ energy metabolism and joint degeneration. Pharmacological treatments, including anti-inflammatory and analgesic agents such as nonsteroidal anti-inflammatory drugs (NSAIDs), glucocorticoids, and biologics, are commonly employed for inflammatory osteoarthropathies and autoimmune-related TMJ diseases such as rheumatoid arthritis. However, these pharmacotherapies have notable limitations, as they often provide only symptomatic relief and fail to halt disease progression. Among the NSAIDs used, drugs such as indomethacin, meloxicam, and celecoxib exert their effects primarily through the inhibition of cyclooxygenase enzymes COX-1 and COX-2, which are involved in the biosynthesis of prostaglandins. Prostaglandins are key mediators of inflammation and pain in joint tissues. Selective COX-2 inhibition by agents like celecoxib can attenuate synovial inflammation and cartilage degradation while minimizing gastrointestinal side effects associated with COX-1 inhibition (62). Despite their analgesic benefits, long-term use of NSAIDs is constrained by adverse effects such as gastrointestinal ulceration, renal dysfunction (63), and cardiovascular risks (64).

Glucocorticoids, by contrast, act through intracellular glucocorticoid receptors to suppress pro-inflammatory gene expression via inhibition of transcription factors such as NF-κB and AP-1. This mechanism reduces the production of cytokines like IL-1β and TNF-α, which are implicated in synovial inflammation and bone resorption (65). Biologics targeting these cytokines, including TNF inhibitors and IL-6 antagonists, represent promising therapeutic options but remain under-evaluated in TMJ-specific contexts.

Beyond these standard anti-inflammatory agents, recent studies have drawn attention to neurotrophic factors such as brain-derived neurotrophic factor (BDNF) and nerve growth factor (NGF), which may contribute to pain modulation by promoting neural plasticity and reducing neuropathic sensitization in chronic TMJ disorders. These agents hold potential to complement traditional therapies by improving both sensory function and patient-reported outcomes, although their clinical applications are still emerging (66).

While pharmacologic treatments can provide symptom control, they do not address the fundamental dysregulations in cellular and energy metabolism that underlie TMJ degeneration (67). This has led to increased interest in surgical interventions, especially in cases of persistent structural joint damage or metabolic dysfunction. Arthroscopic and open surgical procedures, including disc repositioning, joint debridement, and total joint replacement, are employed when conservative measures fail. Arthroscopy is particularly effective for managing internal derangements and synovitis, offering advantages such as reduced morbidity, rapid recovery, and direct visualization of intra-articular pathology (68). However, it is less effective in advanced cases involving ankylosis or severe bone remodeling, where open surgery becomes necessary. Indications for surgery typically include mechanical dysfunction, progressive pain unresponsive to medication, and radiographic evidence of structural deterioration (69).

Conservative treatments, meanwhile, remain central to early-stage management. These include physical therapies such as myofascial release, joint mobilization, and exercise therapy, along with thermal applications (heat/cold), behavioral therapy, and the use of occlusal splints to alleviate masticatory muscle hyperactivity and reduce joint overload. While these approaches improve pain and function, they do not target underlying bioenergetic deficiencies or restore mitochondrial function, which is increasingly recognized as a key factor in TMJ degeneration (19).

Emerging evidence suggests that metabolic dysfunction in TMJ tissues—such as impaired mitochondrial respiration, excessive oxidative stress, and altered glucose utilization—contributes to the pathological changes seen in osteochondral units and synovial tissues. These metabolic disturbances can lead to decreased ATP production, increased reactive oxygen species (ROS), and cellular senescence, ultimately undermining joint integrity and repair. Thus, therapies aimed at correcting energy metabolism hold significant promise (70).

One such intervention involves the intra-articular injection of recombinant human basic fibroblast growth factor (rhFGF). This growth factor promotes fibroblast proliferation and collagen synthesis, contributing to extracellular matrix regeneration and improved joint function. Its capacity to restore synovial homeostasis and support chondrogenesis suggests potential for long-term disease modification (71). Additionally, inducing autophagy in skeletal muscle and periarticular tissues has been shown to enhance energy efficiency and reduce oxidative damage (72). Autophagy, a catabolic process that recycles damaged organelles and proteins, can be pharmacologically stimulated using agents like MYTHO and ROS. These agents inhibit glycolytic flux in skeletal muscle cells, thereby promoting mitochondrial autophagy (mitophagy) and restoring metabolic equilibrium (73). Although promising, the precise regulatory pathways involved—such as AMPK activation or mTOR inhibition—remain under investigation (74).

Another novel therapeutic direction is the use of fibroblastin inhibitors, such as fibroblastin-2, which attenuate abnormal energy metabolism by downregulating NADPH oxidase activity (75). This reduces ROS production and may prevent oxidative damage to cartilage and subchondral bone. While such biologics hold promise, they remain in early experimental stages, and their systemic effects, long-term safety, and specific efficacy in TMD require further validation (76).

Table 2 provides a summarized comparison of the main treatment approaches for TMD, with a particular emphasis on their mechanisms of levels of clinical evidence, therapeutic advantages, limitations, and current validation status.

**Table 2:** Comparative overview of therapeutic strategies for TMD.

Therapeutic strategy	Clinical evidence	Advantages	Limitations	Validation status
NSAIDs	Widely used; supported by clinical trials	Rapid symptom relief; oral availability	GI, renal, CV side effects; no disease modification	Clinically established
Glucocorticoids	Effective in short-term use	Potent anti-inflammatory effects	Long-term toxicity; immunosuppression; not joint-specific	Clinically established
Biologics (TNF/IL-6 inhibitors)	Effective in RA; limited TMD-specific trials	Targeted therapy; may reduce progression	High cost; injection-related risks; limited TMD data	Experimental for TMD
BDNF/NGF-targeting agents	Preclinical and early clinical studies	Potential for chronic pain relief	Safety and long-term effects unknown	Emerging/early-stage
rhFGF (recombinant human FGF)	Animal studies; pilot trials ongoing	Regenerative potential; intra-articular targeting	Local reaction risk; costly production	Experimental
Autophagy inducers	Mostly preclinical studies	Targets core energy imbalance; systemic metabolic improvement	Uncertain human efficacy; systemic metabolic impact	Experimental
Fibroblastin-2 inhibitors	In vitro and animal data	Disease-modifying potential; anti-inflammatory effect	Early-stage research; unknown systemic effects	Preclinical/early-stage
Surgical interventions	Broad clinical experience	Effective for severe degeneration; directly addresses structure	Invasive; postoperative risks; does not reverse metabolic dysfunction	Clinically validated
Conservative therapies	Strong evidence for early-stage TMD	Non-invasive; cost-effective; good for initial stages	Does not address bioenergetic or inflammatory causes	Standard of care

## 6 Summary and outlook

The pathogenesis of TMD is highly complex, with abnormal energy metabolism playing a crucial role in the underlying pathology. Specifically, disruptions in mitochondrial oxidative phosphorylation, glucose utilization, and lipid metabolism within joint tissues contribute to bioenergetic failure and excessive ROS production. These metabolic disturbances lead to changes in local tissue structures, such as the degeneration of articular cartilage, disc displacement, and the formation of adhesions between discs and surrounding musculature. A summary of these relationships is presented in Table 3.

**Table 3:** Specific metabolic dysfunctions and their pathological effects on temporomandibular joint structures.

Metabolic alteration	Mechanism	Pathological consequence
1. Glucose metabolism		
↓G6PD in PPP	↓NADPH→impaired glutathione regeneration→ROS accumulation	Oxidative damage to chondrocytes and cartilage matrix degradation
Hyperglycemia	Impaired proteoglycan and collagen synthesis	Loss of cartilage elasticity and accelerated degeneration
↑Lactate (anaerobic glycolysis)	↓pH→MMP activation (e.g., MMP-1, MMP-13)	ECM breakdown and cartilage erosion
2. Lipid metabolism		
↑ω-6/↓ω-3 fatty acid ratio	↑Pro-inflammatory eicosanoids; ↓anti-inflammatory lipid mediators	Chronic synovitis, chondrocyte apoptosis, and cartilage erosion
↑Cholesterol	Inhibits Notch1 signaling→↑NF-κB and MMPs	Enhanced matrix degradation and inflammation
Estrogen signaling	↑MMP-9, MMP-13 expression in fibrocartilage	ECM breakdown and subchondral bone loss
3. Mitochondrial dysfunction		
Impaired oxidative phosphorylation	↓ATP→disrupted ECM synthesis and ion homeostasis	Chondrocyte dysfunction and cartilage thinning
Mitochondrial ROS overproduction	Oxidative damage to lipids, proteins, and DNA	Inflammation, cellular senescence, and tissue degeneration

Altered energy metabolism impairs the synthesis and turnover of extracellular matrix components, including proteoglycans and collagen, thereby compromising the biomechanical integrity and elasticity of the articular disc and cartilage. These pathological changes ultimately result in TMJ pain and dysfunction.

Targeting abnormal energy metabolism may thus offer a novel therapeutic strategy for preventing or treating TMJ-related diseases. Promising approaches include agents that regulate mitochondrial metabolism, modulate autophagy in chondrocytes and fibroblasts, or restore redox balance through antioxidant pathways. However, a comprehensive understanding of the etiology and pathogenesis of TMJ energy metabolism abnormalities remains lacking. Future research should include molecular and genetic studies to investigate the regulatory networks underlying metabolic dysregulation, as well as clinical trials to assess targeted metabolic therapies. In particular, further exploration is needed into the metabolic behavior of fibroblasts, chondrocytes, and synovial cells under pathological conditions. By clarifying the causes, mechanisms, and therapeutic targets of metabolic disruption, new strategies for the clinical management of TMJ diseases can be developed.
